# Intramolecular circularization increases efficiency of RNA sequencing and enables CLIP-Seq of nuclear RNA from human cells

**DOI:** 10.1093/nar/gkv213

**Published:** 2015-03-26

**Authors:** Yongjun Chu, Tao Wang, David Dodd, Yang Xie, Bethany A. Janowski, David R. Corey

**Affiliations:** 1 Departments of Pharmacology and Biochemistry, 6001 Forest Park Road, Dallas, TX 75390-9041, USA; 2 Quantitative Biomedical Research Center, Department of Clinical Science, 6001 Forest Park Road, Dallas, TX 75390-9041, USA; 3 Simmons Cancer Center, UT Southwestern Medical Center, 6001 Forest Park Road, Dallas, TX 75390-9041, USA

## Abstract

RNA sequencing (RNA-Seq) is a powerful tool for analyzing the identity of cellular RNAs but is often limited by the amount of material available for analysis. In spite of extensive efforts employing existing protocols, we observed that it was not possible to obtain useful sequencing libraries from nuclear RNA derived from cultured human cells after crosslinking and immunoprecipitation (CLIP). Here, we report a method for obtaining strand-specific small RNA libraries for RNA sequencing that requires picograms of RNA. We employ an intramolecular circularization step that increases the efficiency of library preparation and avoids the need for intermolecular ligations of adaptor sequences. Other key features include random priming for full-length cDNA synthesis and gel-free library purification. Using our method, we generated CLIP-Seq libraries from nuclear RNA that had been UV-crosslinked and immunoprecipitated with anti-Argonaute 2 (Ago2) antibody. Computational protocols were developed to enable analysis of raw sequencing data and we observe substantial differences between recognition by Ago2 of RNA species in the nucleus relative to the cytoplasm. This RNA self-circularization approach to RNA sequencing (RC-Seq) allows data to be obtained using small amounts of input RNA that cannot be sequenced by standard methods.

## INTRODUCTION

RNA sequencing (RNA-Seq) has become a widely used tool for investigating gene expression (1). Millions of sequence ‘reads’ in combination with bioinformatic analysis and experimental validation can provide new insights into fundamental cellular processes. The usefulness of RNA-Seq, however, is often limited by the amount of input RNA needed to yield meaningful data.

RNA-Seq can be used to analyze both long RNA and small RNAs. For the sequencing of long RNA fragments (>200 bases), the most sensitive methods may allow researchers to study single cell transcriptome and require as little as 10–100 pg of total RNA as input (2–5). Standard long RNA sequencing methods often use random priming to generate reads across the entire length of all transcripts under study (6,7). Random priming, however, is not an option for sequencing small RNAs because they are unlikely to yield DNA sequences of sufficient length to be mapped uniquely within a genome. To sequence small RNA (<200nt), including miRNAs, endogenous siRNAs, piRNAs, and heavily-fragmented long RNAs, library preparation generally requires ligation of short sequences to the 3′- and 5′-ends of the RNAs to serve as hybridization sites for standardized PCR primers (8,9).

Reliance on intermolecular ligations for a critical step in RNA-Seq can be problematic. Introduction of two primer binding sites requires two successful intermolecular ligation steps and increases the minimum amount of input small RNA required. In the case of the widely used Tru-Seq small RNA preparation protocol, 1–10 μg of total RNA is recommended to obtain sufficient small RNA as input for miRNA sequencing (http://support.illumina.com/sequencing). When total RNA is used as input for miRNA sequencing, 1 μg of total RNA is required (http://support.illumina.com/sequencing). Intermolecular ligations are also sensitive to sequences close to the RNA termini (9). This sensitivity can generate sequencing biases (9) and structure at the 3′ terminus of RNA can cause some sequences to be under-represented (10).

For some applications, obtaining ≥1 μg of total RNA is difficult and sequencing small RNA will be challenging. These applications include analysis of small RNA from: (i) extracellular RNA (11); (ii) relatively small numbers of cells or single cells; (iii) scarce clinical samples; (iv) RNA purified from cellular compartments such as mitochondria (12) or nuclei and (v) RNA isolated after immunoprecipitation protocols like CLIP-Seq (13,14). Our goal was to (1) develop a straight-forward methodology that could be readily adopted by researchers accustomed to standard RNA-Seq protocols and platforms and (2) achieve higher sensitivity for miRNAs and other small (<100 nucleotides) RNAs and RNA fragments.

**Figure 1.**
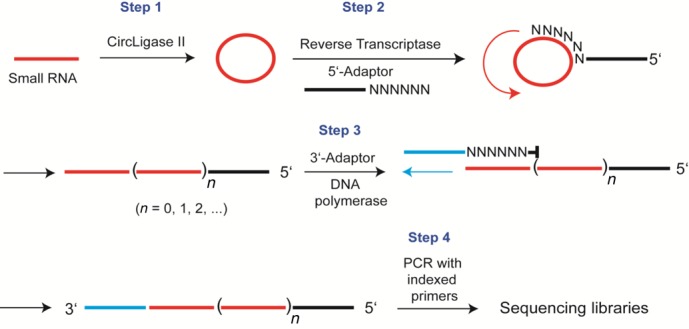
Scheme showing RC-Seq library preparation.

To accomplish this goal, we exploited the principle that intramolecular reactions are more favorable than intermolecular reactions by developing a sequencing methodology that uses RNA self-circularization (RC-Seq) (Figure 1). A basic principle of chemical recognition and reactivity is that, in the absence of steric constraints, intramolecular associations proceed more rapidly than analogous intermolecular processes (15–18). The rate of DNA (19–21) or RNA (22) ligations is much faster and more efficient when the effective concentration of reactive termini is increased.

In our protocol, we circularize the RNA template through an intramolecular ligation. This circularization allows us to prime cDNA synthesis with tagged random primers that bind the RNA template by base-pairing. These steps avoid the need to attach adaptor oligonucleotides to the RNA through intermolecular ligations. By substituting intra- for intermolecular ligation, we increased sensitivity of RNA sequencing, simplified the library purification steps and enable applications that could not be accomplished otherwise. Using this method, we show that we can obtain RNA sequencing data from nuclear samples after UV-crosslinking to protein and immunoprecipitation for samples where the use of standard methods did not yield data.

## MATERIALS AND METHODS

### Cell culture and obtaining synthetic oligonucleotides

T47D cells (American Type Culture Collection, ATCC) were maintained in RPMI-1640 media supplemented with 10% (v/v) fetal bovine serum (FBS), 0.5% (w/v) nonessential amino acids, 0.4 units/ml bovine insulin (all reagents from Sigma). Cells were cultured at 37°C and 5% (v/v) CO_2_. All synthetic RNAs, primers for generating cDNA and PCR primers were obtained from Integrated DNA Technologies (IDT) and purified by polyacrylamide gel electrophoresis (PAGE).

### PAR-CLIP sample preparation

T47D cells were incubated in fresh media containing 4-thiouridine (Sigma) at 100 μM. Medium was removed 14 h later and cells were washed once with Dulbecco's phosphate buffered saline (Sigma) and UV-irradiated at 365 nm with an energy of 300 mJ/cm^2^ on ice. Nuclei were isolated by first incubating the cells in hypotonic lysis buffer (10 mM Tris•HCl pH 7.4, 10 mM NaCl, 3 mM MgCl_2_, 0.5% NP-40, 1× complete protease inhibitor (Roche), 0.5 mM dithiothreitol (DTT) and 50 U/ml Promega RNase-In) for 5 min on ice. The supernatant was removed following centrifugation at 500 × g for 5 min at 4°C. This process was repeated once. The crude nuclei were further washed twice with this hypotonic buffer to yield pure nuclei. The pure nuclei were then suspended in nuclear lysis buffer (150 mM KCl, 20 mM Tris•HCl 7.4, 1.5 mM MgCl_2_, 0.5% NP-40, 1× complete protease inhibitor, 0.5 mM DTT and 50 U/ml Promega RNase-In) for 10 min on ice. After vigorous vortexing and pipetting, nuclei were subjected to three freeze-thaw cycles using liquid nitrogen and a 22°C water bath. The mixture was then subjected to short pulse sonication using an Ultrasonic Homogenizer (20% power for 30 s , Model 150V/T, Biologics, Inc.), followed by DNase I (Worthington) treatment at 37°C for 15 min. Insoluble material was removed by centrifugation at maximum speed for 15 min at 4°C. Nuclear extracts were quickly frozen in liquid nitrogen and stored at −80°C.

The Ago2 immunoprecipitation and clipped RNA isolation were carried out based on the original PAR-CLIP protocol (14) with two exceptions (i) RNase I (Life Technologies) was used instead of RNase T1 to avoid potential sequence biases generated from using RNase T1 and (ii) an anti-Ago2 antibody (Sigma, 11A9), that recognizes endogenous Ago2, was used. The final recovered RNA was quantitated with RiboGreen RNA assay kit (Life Technologies).

### RNA circularization

RNA, including synthetic RNA or clipped RNA were circularized with CircLigase™ II ssDNA Ligase (Epicentre) at 60°C for 1 h in a 20 μl reaction volume containing 2 μl 10× reaction buffer, 1 μl 50 mM MnCl_2_ (Epicentre), 4 μl 5 M Betaine (Epicentre) and 1 μl ligase. To remove the remaining linear RNA, 2.3 μl of 10× RNase R buffer (Epicentre) and 1 μl of RNase R (20 U, Epicentre) was added to the reaction mixture. The RNase R digestion was carried out at 37°C for 10 min. After the digestion, an oligo purification column (Zymo Research, Oligo Clean & Concentrator) was used to isolate the circularized RNA following the manufacturer's instructions. Purified RNA was eluted with nuclease-free water.

### RC-Seq library preparation

The first step in library preparation is generation of the complementary DNA (cDNA) strand from the circularized RNA. To generate the cDNA strand, we mixed a circularized RNA solution with 0.5 μl cDNA primer (GACGTGTGCTCTTCCGATCTNNNNNN, 100 μM), 1 μl 10 mM dNTP solution (containing 10 mM dATP, 10 mM dGTP, 10 mM dCTP and 10 mM dTTP) and H_2_O to a total of 12 μl. Following heating of the solution at 65°C for 5 min on a thermal cycler, it was cooled directly on ice for at least 1 min. To this solution was added 4 μl 5× Superscript II reaction buffer, 2 μl 0.1M DTT, 1 μl RNase-Out and 1 μl Superscript II (Life Technologies). The solution was mixed gently and placed on a thermal cycler at 25°C for 15 min, then 42°C for 30 min. Next, 2μl of 1 M KOH was added solution and heated to 95°C for 15 min after which the solution was cooled to room temperature and neutralized by 2 μl 1 M HCl.

The cDNA was column-purified (Zymo Research, DNA Clean & Concentrator) and eluted with 17.5 μl H_2_O. To this cDNA solution was added 0.5 μl 0.1 M DTT, 1 μl 10 mM dNTP, 0.5 μl 100 μM 3′-end extension primer (ACACGACGCTCTTCCGATCTN_*n*_/3Phos, *n* = 6 or 8), 5 μl 5× Buffer 2 (New England Biolabs, NEB) and 0.5 μl Klenow DNA polymerase I (NEB). The extension reaction was carried out at 25°C for 15 min, and then at 95°C for 3 min to deactivate the enzyme. The tagged cDNA was purified again with a column (Zymo Research, DNA Clean & Concentrator) and eluted with 23.5 μl H_2_O. To the purified cDNA solution, 25 μl Failsafe PCR Premix E (Epicentre), 0.5 μl forward PCR primer (50 μM, Illumina), 0.5 μl reverse PCR primer (50 μM, Illumina, indexed), and 1 μl Failsafe PCR enzyme (Epicentre) were added. The mixture was first heated at 95°C for 1min, followed by 12–24 cycles (depending on the quantity of RNA input) of 95°C for 30 s, 55°C for 30 s and 68°C for 3 min with a final extension step at 68°C for 7 min. The crude PCR product was purified by Agencourt AMPure XP magnetic beads (Beckman Coulter) using a 1:1 volume ratio. Final PCR product was eluted with H_2_O and analyzed by Bioanalyzer for library size distribution. The library was then quantitated by PicoGreen Assay and sequenced with Illumina HiSeq2000 in a paired-end mode (2 × 100nt).

### RC-Seq library data analysis

Each pair of the raw reads was merged to obtain the full-length sequence of the original cDNA molecules using the program FLASH. The minimum overlapping length was set at 10nt. The merged paired-end reads were kept in one file, while the unmerged paired-end reads were kept in two different files. Next, each merged paired-end read as well as each first read in the unmerged reads file underwent repeating unit extraction using a Perl script (part of the developed package). In this script, maximum error number is set at 10% of the length of a repeating unit. After the repeating unit is identified for each read, a read expansion script is used to expand the repeating unit by moving one base at a time from its 5′-end to its 3′-end so the number of reads generated in the group is equal to the number of bases of the repeating unit. Each read in the group was then aligned to hg19 using TopHat2 with the default parameters (maximum two errors). For CLIP-Seq data, each read was aligned to a reference transcriptome (hg19) generated within the TopHat2 program. A SAM output file for accepted alignments was obtained. For a group of expanded reads, only uniquely aligned derivatized reads were kept. Next, an algorism to examine if all the unique alignments were converged to the same genomic/transcriptome location was executed. If aligned, the best alignment (with highest alignment score or lowest errors) is chosen as the read representing the original RNA sequence.

### Implementation of scripts used in RC-Seq data analysis

Three separate perl scripts used for read repeating unit extraction (script-1), repeating unit expansion (script-2) and finding the original RNA sequence (script-3) will be made available at: http://www4.utsouthwestern.edu/coreylab/protocols.html. Script-1 uses the raw read file containing merged paired-end reads as the input. The output file of script-1 is the input file of script-2. Script-3 uses alignment output SAM file as the input and produces sorted BAM file for downstream analysis.

### Expanded alignment approach: validation by simulation

To examine whether the expanded alignment approach is valid in aligning the sequencing data to the correct positions in the genome, an *in silico* simulation was carried out. In the simulation, five groups of reads of different lengths were generated, 20 nt, 40 nt, 60 nt, 80 nt and 100 nt. For each group, 5000 reads were randomly selected computationally. The original genomic location of each read was recorded during generation. For the traditional alignment method, these 5000 reads were aligned to hg19 using TopHat2 and only uniquely aligned reads and their alignments were retained. By comparison with their original genomic locations, the percentages of correctly uniquely aligned reads were calculated. For the expanded alignment approach, each of the 5000 reads was circularized to mimic the repeating pattern of the RC-Seq sequencing data. Then the expanded alignment method was employed to align the generated reads to the genome. Similarly, the percentages of correctly and uniquely aligned reads were calculated.

### Cluster identification by MiClip package

We used MiClip to find the RNA binding sites of the Ago2 protein. The MiClip package has been reported and is publicly available at CRAN (http://cran.r-project.org) (23). We first pooled the single-end alignment data for all replicates of the same experimental condition. We then ran the MiClip algorithm with characteristic mutations set to T→C mutations for PAR-CLIP, and bin size set to 10bp, to detect the significant binding sites of the Ago2 proteins. The same set of parameters was used for all PAR-CLIP samples reported in this study.

## RESULTS

### Intramolecular circularization using Circligase II

Our sequencing method is based on circularizing input RNA to avoid the need to introduce primer binding sites using an intermolecular ligation step. We chose Circligase II for the intramolecular ligation (Figure 1, step 1) because it is a thermostable enzyme that efficiently catalyzes circularization of DNA or RNA templates that possess 5′-phosphate and 3′-hydroxyl groups (24,25). Circularization was carried out at 60°C to minimize structure at the termini that might prevent efficient ligation of sequences.

**Figure 2.**
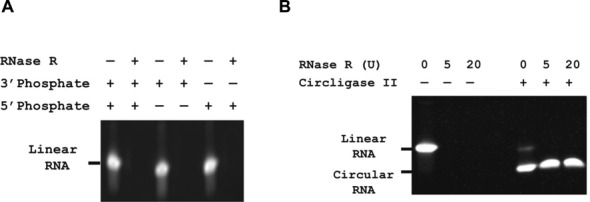
Characterizing action of RNase R and Circligase II. (**A**) RNase R digests single stranded RNA efficiently, regardless of phosphate location. Linear substrate RNA with either 3′ or 5′ phosphate in the presence or absence of RNase R. (**B**) RNase R digests linear, but not circular, RNA. Circularized RNA was obtained by treating linear RNA (5′-phos-UUUGCAAAGAUAGUUGUGCUU) with Circligase II for 1 h at 60°C. RNA samples, linear or circular, were treated with varying amounts of RNase R (0, 5 or 20 units) at 37°C for 15 min. Samples were loaded on 15% Tris/borate/EDTA (TBE)–urea gel and stained with Sybr Gold for visualization.

We optimized ligation conditions using a synthetic 21 base oligomer. We added Circligase II to circularize the RNA followed by RNase R to remove residual linear RNA (Figure 2). RNase R (26) was chosen because it efficiently degrades single-stranded linear RNA with almost complete discrimination against cleavage of circular RNA. Removing short single-stranded linear RNA simplifies subsequent purification and analysis of amplified products. Short linear RNA will often be a poor template for randomized primers because the products lack full-length RNA sequence information. Furthermore, single-stranded RNA will compete with circular RNA during each step of library preparation and sequencing, reducing sequencing depth.

The presence or absence of terminal phosphate groups can affect the activity of nucleases and the efficiency of template preparation. miRNAs have 5′ phosphate groups while other input RNAs could have 3′ phosphate groups. It is important, therefore, to understand the capacity of RNase R to degrade RNAs with differing phosphate groups. To ensure that RNase R would be capable of fully digesting all the RNA in our input samples, we examined digestion of RNA with phosphate groups at either or both the 3′ and 5′ termini. RNase R efficiently degraded RNA regardless of phosphate location (Figure 2A), suggesting that RNAse R is a good choice for removing residual linear RNA.

We then tested the conversion of linear to circularized RNA by Circligase II in the presence or absence of RNase R. We found that we could achieve a conversion rate to a single circularized product of >80% (Figure 2B). Addition of RNase R to circularization reactions removed remaining linear RNA, preventing the linear RNA from interfering with subsequent steps.

By contrast, using the best reagents available for RNA adaptor ligation (essentially the same as what provided in the Tru-Seq small RNA preparation kit), we carried out the ligation reaction at 25°C for 2 h and observed a conversion rate of only ∼49%, with at least one by-product formed, likely a concatamer (27) (Supplementary Figure S1A). Lowering the reaction temperature to 15°C and extending the reaction time to 16 h resulted in a cleaner product mixture but reduced conversion rate at ∼25% (Supplementary Figure S1B).

### Using circularized template to generate cDNA libraries

To prime reverse transcription of the circularized template and install a 5′ recognition sequence for subsequent PCR, we mixed the template with tagged random primers and allowed them to hybridize by Watson–Crick base-pairing. The tag enables sequencing by Illumina instruments without changing standard sequencing protocols.

To identify the best length of randomized bases we tested six, eight, and ten base random segments. We chose to use randomized hexamers for subsequent experiments because we observed that increasing the size of the randomized region from six to ten did not increase the efficiency of reverse transcriptase. After hybridization, the circularized RNA/primer complex was incubated with Superscript II reverse transcriptase to generate cDNA (Figure 1, step 2).

**Figure 3.**
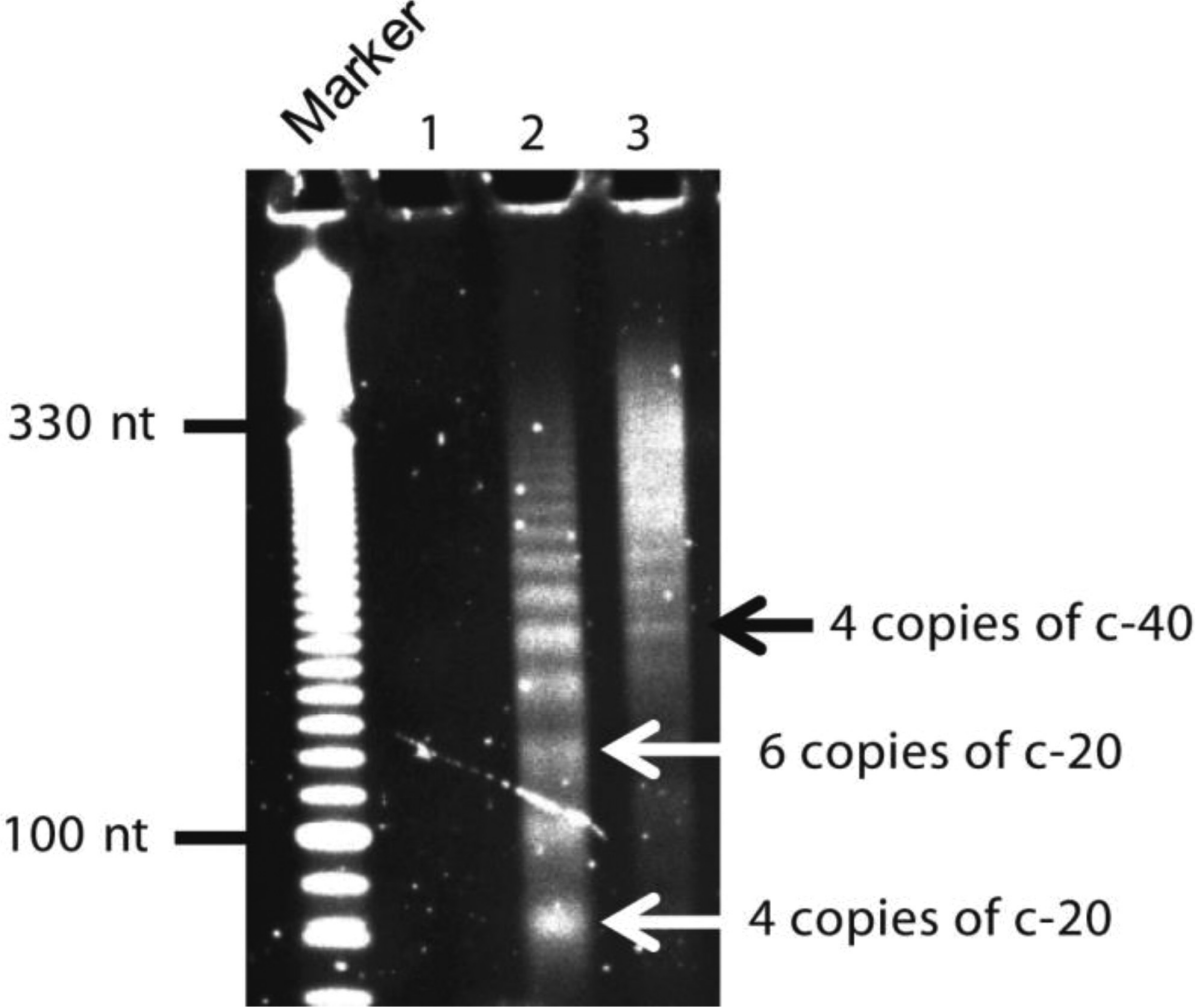
Evaluating reverse transcription efficiency using circular templates of 20 nt or 40 nt randomized RNA. Random hexamers were used to prime reverse transcription (RT). Lane1: product from RT reaction with no RNA. Lanes 2 and 3: products from RT reaction with circular templates of 20 nt and 40 nt randomized RNA, respectively. Bands corresponding to four or six copies of 20 nt circular RNA (c-20) and four copies of 40 nt circular RNA (c-40) are noted. Following RT reaction, cDNA was purified and loaded on a 6% TBE–urea gel then stained with Sybr Gold for visualization.

Because the RNA template is a circle, reverse transcription proceeds by rolling-circle amplification (RCA) in which multiple copies of the template are transcribed (24,25). To gauge the dependence of library preparation on template size, we carried out reverse transcription using an equal amount of randomized 20 nt and 40 nt RNA circles using random hexamer primers (Figure 3). Both templates were amplified in multiple copies and efficiencies were similar.

After obtaining linear cDNA, we hybridized a tagged random primer and added DNA polymerase to extend the DNA strand. This polymerization creates a product with two primer recognition sites that could be used for PCR (Figure 1, step 3). The tagged primer was blocked at the 3′ position so that it would not be extended. Instead, the cDNA is extended to contain the sequence complementary to the primer. The cDNA from our method is generally greater than 100 bases, allowing us to purify the cDNA using magnetic beads or silica columns. These purification steps are advantageous because they are fast and simple relative to PAGE purification.

We then performed one round of PCR on the purified cDNA with one primer binding the 3′ tag, and a second primer (indexed) binding the 5′ tag. The resultant library was subjected to single-end or paired-end sequencing (Figure 1, step 4). Depending on the measured quantity of starting RNA being used, the PCR cycles could vary from 12 to 24 cycles with the latter reserved for picograms of starting RNA. The amplified DNA library for sequencing is greater than 200 bp in length and was purified conveniently using AMPure XP magnetic beads.

### Computational analysis of sequencing data from RC-Seq

**Figure 4.**
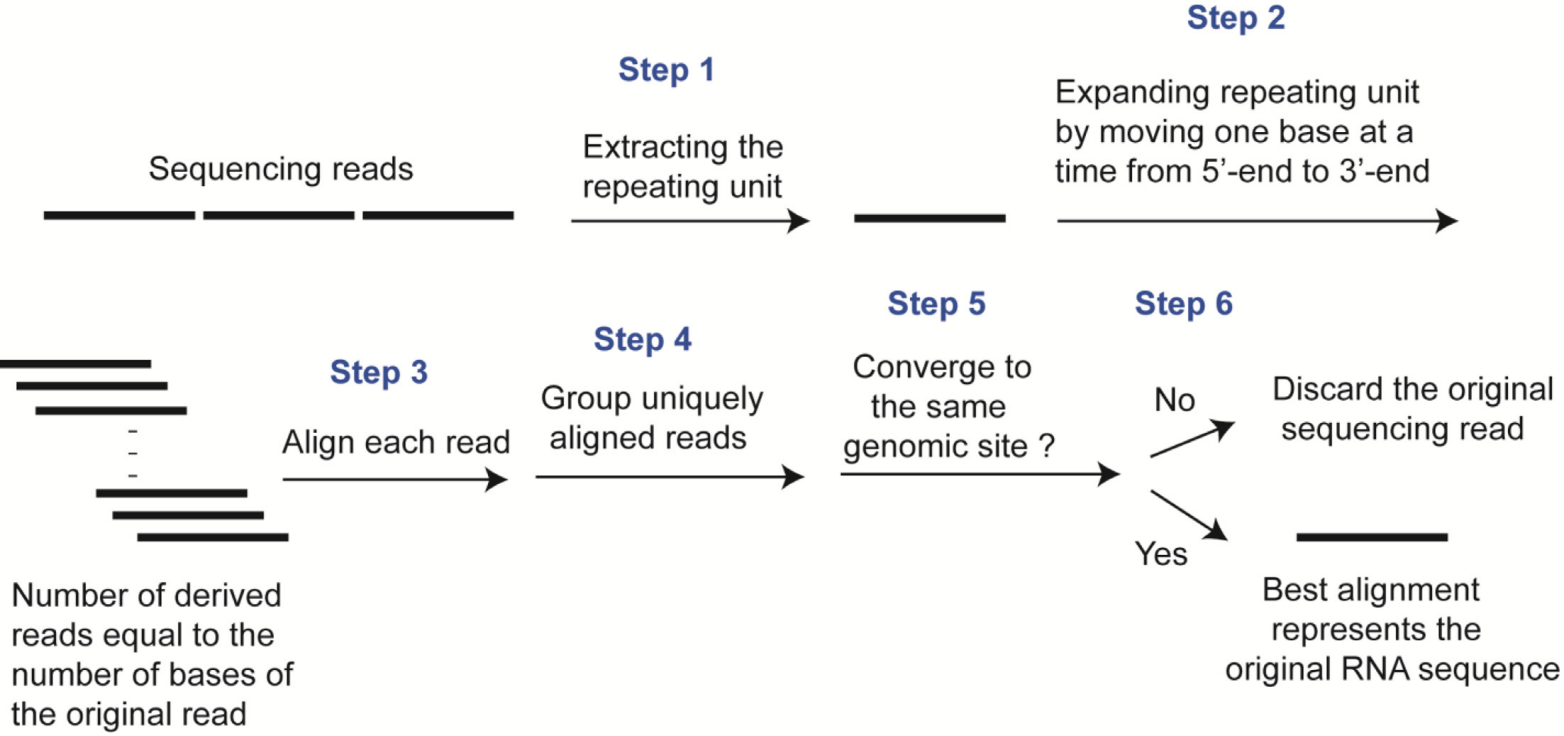
Processing of RC-Seq data. Expanded alignment approach is used in the sequencing data alignment. Only uniquely aligned reads are kept for downstream analysis.

After library preparation we performed RNA-Seq and analyzed the resulting reads. Existing software was not able to efficiently locate the original sequences because our template is circular and subject to rolling circular amplification (RCA) (24,25). We anticipated that we would observe multiple copies of many small RNA sequences within the cDNA and dealt with this outcome by developing protocols for analysis that can be downloaded and run on any operating system (Figure 4).

All the reads contained repeating units, and our first step was to identify the repeating unit as a single sequence (Figure 4, step 1). Depending on where the tagged random hexamer primers hybridize, the repeating unit could differ even if derived from the same parent sequence. To recover the original RNA fragment sequence we computationally shifted from 5′ to 3′ ends in one base increments to create a family of sequences (Figure 4, step 2). Each family member was tested for its ability to align with a reference genome. The family member that was both uniquely aligned and possessed the highest alignment score was determined to represent the original RNA sequence (Figure 4, steps 3–6).

**Figure 5.**
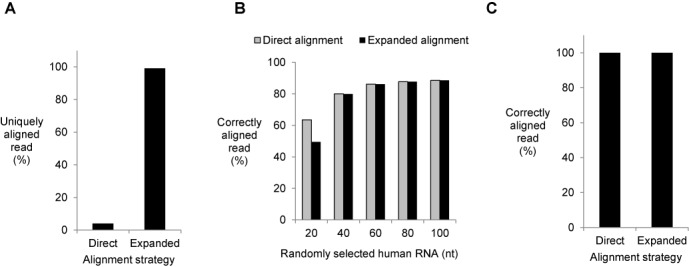
Evaluating data processing efficiency of RC-Seq by simulation. (**A**) The problem: the direct alignment approach does not efficiently align sequencing data generated from RC-Seq. By contrast the expanded alignment approach aligns >99% of sequencing reads. 10 ng of partially randomized synthetic 40 nt RNA mixture was used as input to generate the library (RNA: 5′-phos-NNNNNNNNNNUGAGGUAGUAGGUUGUAUAGNNNNNNNNNN-3′). (**B**) Comparing the efficiency of alignment between direct alignment and expanded alignment approaches with randomly selected short RNA sequences as input. Each read was aligned to human transcriptome. Five different groups of reads, 20 nt, 40 nt, 60 nt, 80 nt and 100 nt, were used in the simulation. For each group, 5000 reads were randomly selected from human genome (hg19) and their original genomic locations were pre-recorded to identify correct alignment. (**C**) Human miRNAs are mapped correctly by both direct alignment and expanded alignment approaches. Human miRNA reference sequences were extracted from miRBase for alignment.

Because the method requires sequencing tandem RNAs, the output sequences are repetitive and cannot be mapped to reference sequences using the standard, direct alignment approach. We used RC-Seq data obtained from partially randomized synthetic 40 nt RNA mixture (with 10 randomized sequence at each terminus) to test the efficiency of standard direct alignment approach and found that only ∼3% of the reads could be aligned (Figure 5A). Using expanded alignment approach described above (Figure 4) we achieved 99% correct alignment of those reads (Figure 5A). Since there was no reference dataset to align to, we counted a read as a mapped read when it contained a defined single sequence (20 nt) from positions 11 to 30 as those found in original RNAs.

We carried out a computational simulation to validate our approach to regenerate the original RNA sequence and subsequently identify its origin within an actual genome. In the simulation, five groups of linear RNA with different sizes (20 nt, 40 nt, 60 nt, 80 nt and 100 nt) were randomly chosen from human genome hg19 and their original locations were recorded. We then used standard alignment program TopHat2 (28) to identify RNA sequences that could be uniquely aligned within hg19 as a benchmark. Using direct alignment approach 60% of randomly selected 20 nt RNA were uniquely and correctly aligned to their original genomic locations (Figure 5B). With RNA size increasing to 40 nt or longer, 80% or more of RNA at each group were correctly aligned.

We then computationally circularized each of these RNAs to mimic the output of RC-Seq and applied the expanded alignment approach. We found that circular random sequences can be uniquely and correctly aligned to their original locations with percentages close or equal to those from the linear RNA of analogous size (Figure 5B). This finding suggested that our computational strategy could overcome the challenge posed by RNA circularization and repeating sequences.

Since miRNAs are an important biological small RNA, we also carried out a simulation for every human miRNA following the strategy described above for both linear and circularized miRNAs (Figure 5C). As we had observed for genome fragment sequences, using miRBase as the database for alignment we found that each miRNA was correctly aligned regardless of whether they were in linear or circularized forms. The data from these simulations using genome fragments and miRNAs suggest that unique RNA sequences (>20 nt) within circular templates can be correctly mapped to their original genome location.

### Sensitivity and possible sequencing biases of RC-Seq

We first tested our experimental and computational protocols using a synthetic forty base RNA template containing ten randomized bases at each terminus. This RNA template was commercially synthesized and it is possible that at certain randomized positions four RNA bases (A, G, C, U) are not equally represented. To gauge the sequencing sensitivity of RC-Seq relative to standard methods we prepared a series of RNA-Seq libraries in parallel using commercially available Tru-Seq small RNA preparation kit and RC-Seq with RNA template quantities varying from 100 to 0.1 ng.

**Figure 6.**
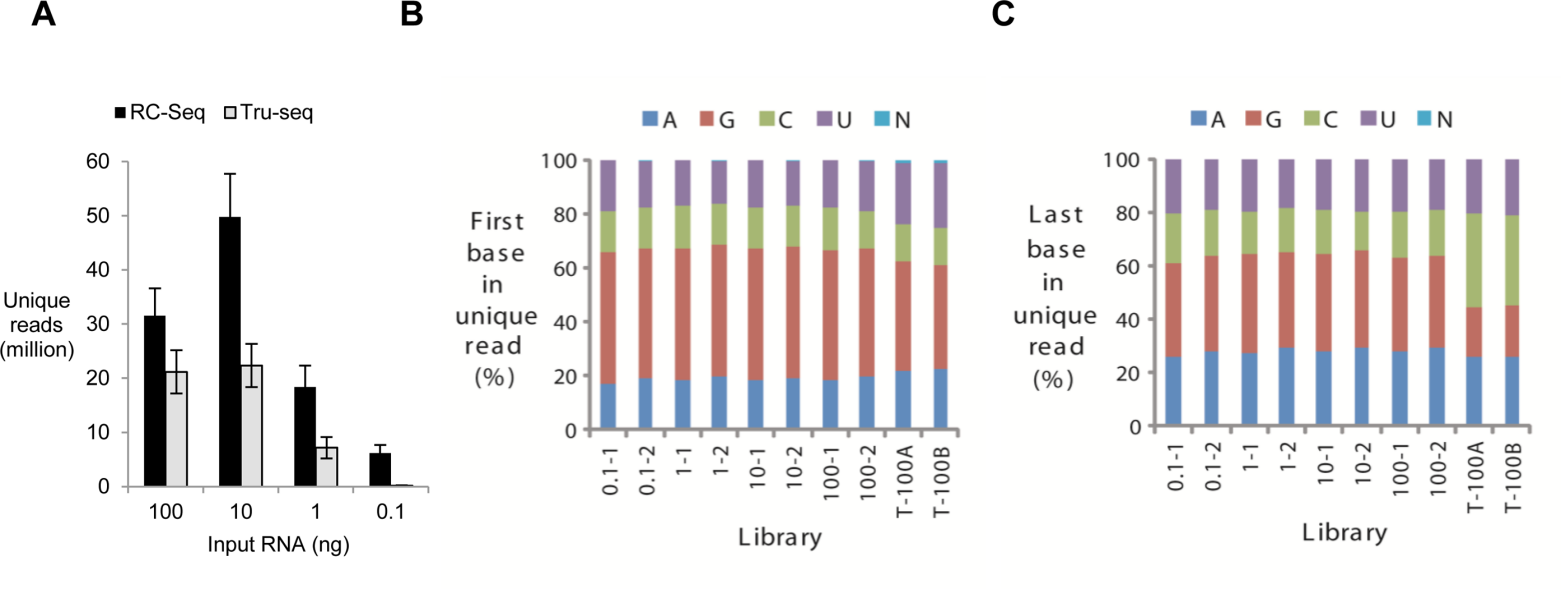
Evaluating the sensitivity and accuracy of RC-Seq. (**A**) Generation of unique reads by RC-Seq and Tru-Seq as a function of varying input quantities of a synthetic 40 base RNA substrate (5′-phos-NNNNNNNNNNUGAGGUAGUAGGUUGUAUAGNNNNNNNNNN-3′). Tru-Seq libraries were prepared with Illumina Tru-Seq small RNA preparation kit. The PCR cycles were the same for RC-Seq and Tru-Seq with the same amount of starting RNA. (**B**) Histogram representing the profile of the base (A, C, G or U) at the 5′-end of unique reads. (**C**) Histogram representing the profile of the base at the 3′-end of unique reads. T-100A and T-100B stand for two replicate data generated from 100 ng of RNA input using Tru-Seq small RNA preparation kit.

We consistently observed a significant increase in unique read numbers for RC-Seq over Tru-Seq when the same amount of starting RNA was used (Figure 6A). For example, we obtained ∼20 million of unique reads for Tru-Seq with 100 ng RNA input. By contrast, RC-Seq generated ∼50% more unique reads using the same amount of input. Although Tru-Seq was still capable of generating multimillion of unique reads with input quantity reduced from 100 ng to 10 ng or 1 ng, it essentially reached its sensitivity limit when the input was <1 ng (Figure 6A). On the other hand, RC-Seq can still yield ∼5 million unique reads with just 100 pg of RNA input (Figure 6A). This data demonstrate that RC-Seq has the potential for significantly greater sequencing sensitivity than Tru-Seq.

To confirm that cDNA library was indeed generated from original RNA input, not from possibly contaminated reagents used during the RC-Seq library preparation, we prepared libraries with no input RNA and RNA of 10 ng of 40 nt randomized sequence side by side and resolved them on an agarose gel. We observed a single band at low molecular weight region (<100 bp) in the "no input RNA" control library, likely a primer–dimer by-product (Supplementary Figure S2). The lane of the library made with 10 ng RNA input displayed expected high molecular weight product. These data confirm the specificity of RC-Seq in generating useful reads from original RNA input molecules.

Ligations may differ in efficiency depending on the identity of terminal bases, creating a bias during library preparation. To investigate possible sequencing biases caused by using CircLigase II, we analyzed the base profile of the first or the last base in each RC-Seq library. At 5′-end, we found that G was the most prevalent base, followed by A, C and U. G was also the major base at 5′-end of Tru-Seq libraries (Figure 6B). At 3′-end for RC-Seq libraries, A and G were more prevalent than C and U (Figure 6C). The Tru-Seq libraries displayed different profile at 3′-end, with C being the most frequent base, followed by A, then G and U (Figure 6C). The profile data suggests that CircLigase II and T4 RNA ligase may have different 3′ base preferences for ligation.

### Nuclear Ago2 PAR-CLIP

To test our methodology in a demanding application, we applied it to the analysis of human nuclear Argonaute 2 (Ago2)-associated RNA obtained from photoactivatable-ribonucleoside-enhanced crosslinking and immunoprecipitation (PAR-CLIP) (14). In PAR-CLIP, cells are grown in the presence of 4-thiouridine (4-SU) to produce labeled RNA. Exposure to UV light at 365 nm specifically crosslinks the 4-SU to associated proteins inside cells. After cell lysis, treatment with RNase followed by immunoprecipitation allows isolation of protein-bound fragmented RNAs for sequencing. The introduction of 4-SU can significantly increase the likelihood of T to C mutations within sequencing reads. Observation of these mutations within a group of reads with similar genomic coordinates suggests a true protein-binding site nearby.

CLIP-Seq can be used to analyze fragmented cellular RNA or miRNAs. Unlike miRNAs, which contain a 5′ phosphate and a 3′ hydroxyl group, RNA fragments may lack these features, preventing their amplification. To ensure that all RNA could be amplified regardless of origin, we used T4 polynucleotide kinase because the enzyme removes 3′ phosphates while adding 5′ phosphates.

A successful PAR-CLIP experiment requires a robust and specific antibody. We used 11A9, a well characterized anti-Ago2 antibody and used by others for CLIP-Seq (29,30). Nevertheless, we still carried out experiments to confirm its specificity (Supplementary Figure S3). When we used 11A9 to pull down radiolabeled protein-RNA complex from crosslinked nuclear lysate, we observed a major band at 110 kDa resolved on SDS-PAGE (Supplementary Figure S3A). Another anti-Ago2 antibody 4G8, which is commonly used for immunoprecipitation, did not pull down the crosslinked complex. If the band at 110 kDa is indeed the Ago2-associated RNA complex, we would expect to see a strong band at ∼100 kDa in 11A9 or 4G8 lanes, corresponding to the non-bound Ago2 protein itself, which should migrate faster than an Ago2-RNA complex. This is because only a small percentage of Ago2–RNA complex can be experimentally crosslinked by UV light and most of Ago2 protein stays in its free-form after separation by SDS-PAGE.

Using the same cellulose membrane, we analyzed the presence of Ago2 by western blot and observed a strong band at 100 kDa in both 11A9 and 4G8 lanes, corresponding to free-form Ago2 (Supplementary Figure S3B). Additionally, when RNA and Ago2 were not crosslinked with UV light, no bound RNA complex was detected (Supplementary Figure S3C). These data suggested that this band (∼110 kDa) contained Ago2 and was the Ago2-RNA crosslinked complex. Sequencing of the isolated RNA revealed substantial miRNA reads in the dataset (data not shown), further confirming that the 110 kDa band is Ago2-associated.

**Figure 7.**
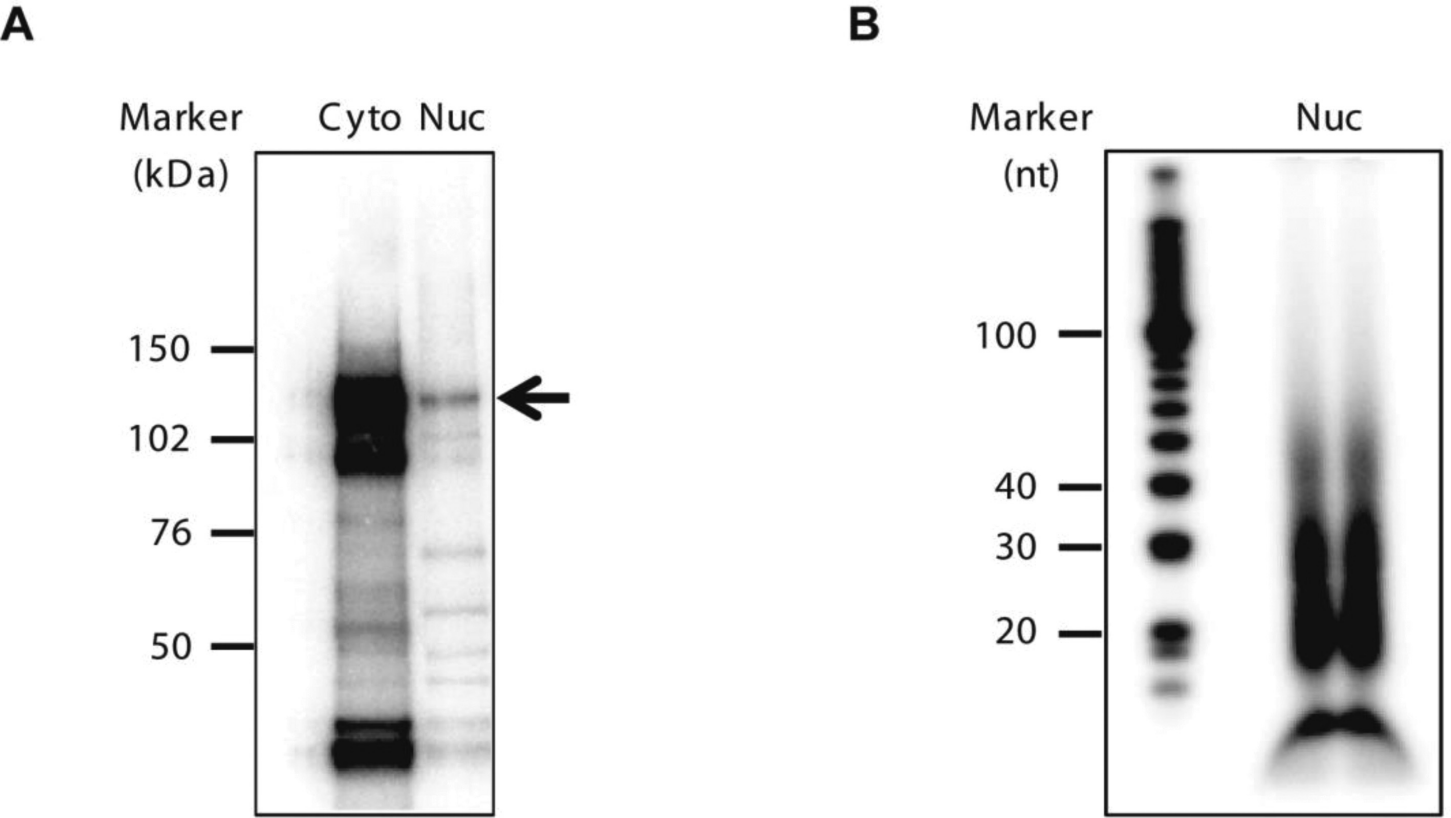
Isolated Ago2-RNA complex and RNA species following a PAR-CLIP. (**A**) Autoradiography of ^32^P-labeled RNA–protein complexes resolved by SDS-PAGE (4–12% gradient gel). Arrow indicates the expected Ago2–RNA complex. Ago2 PAR-CLIP was carried out using cytoplasmic (Cyto) and nuclear (Nuc) cellular fractions, obtained from the same number of cells. (**B**) Autoradiography of ^32^P-labeled RNA isolated from two nuclear (Nuc) Ago2 PAR-CLIP samples, resolved on a 10% TBE–urea gel.

PAR-CLIP of these nuclear samples is particularly challenging because much less RNA is associated with Ago2 in nucleus than that in cytoplasm (Figure 7A). PAR-CLIP employs a nuclease digestion step to remove RNA not associated with protein, leaving only small RNA fragments for analysis. Because of these limitations, after immunoprecipitation, gel purification, proteinase K treatment and ethanol precipitation, we recovered limited quantities of RNA as input for library preparation (<1 ng) even when starting with close to one billion T47D breast cancer cells. The RNA we captured at the end of our procedures typically ranges from 20 nt to 50 nt in size (Figure 7B).

### Analysis of PAR-CLIP data from RC-Seq

Upon obtaining the RNA from nuclear Ago2 PAR-CLIP, we made repeated efforts to prepare sequencing libraries using standard Tru-Seq protocols (Supplemental Figure S4). In seven independent ligation experiments, we did not detect ligated products between input RNA and adaptor oligonucleotide. Including more stringent treatment with polynucleotide kinase to remove 3′ phosphates or tripling the amount of input nuclear lysate for immunoprecipitation did not afford detectable product (Supplemental Figure S4A–C). Even though no ligated product was apparent, we attempted to prepare cDNA libraries. We observed no cDNA upon gel electrophoresis after six attempts using the Tru-Seq protocol for small RNA library preparation (Supplemental Figure S4D and E). Bioanalyzer examination showed no visible product peaks. These outcomes emphasized the need for a new approach for library preparation.

**Figure 8.**
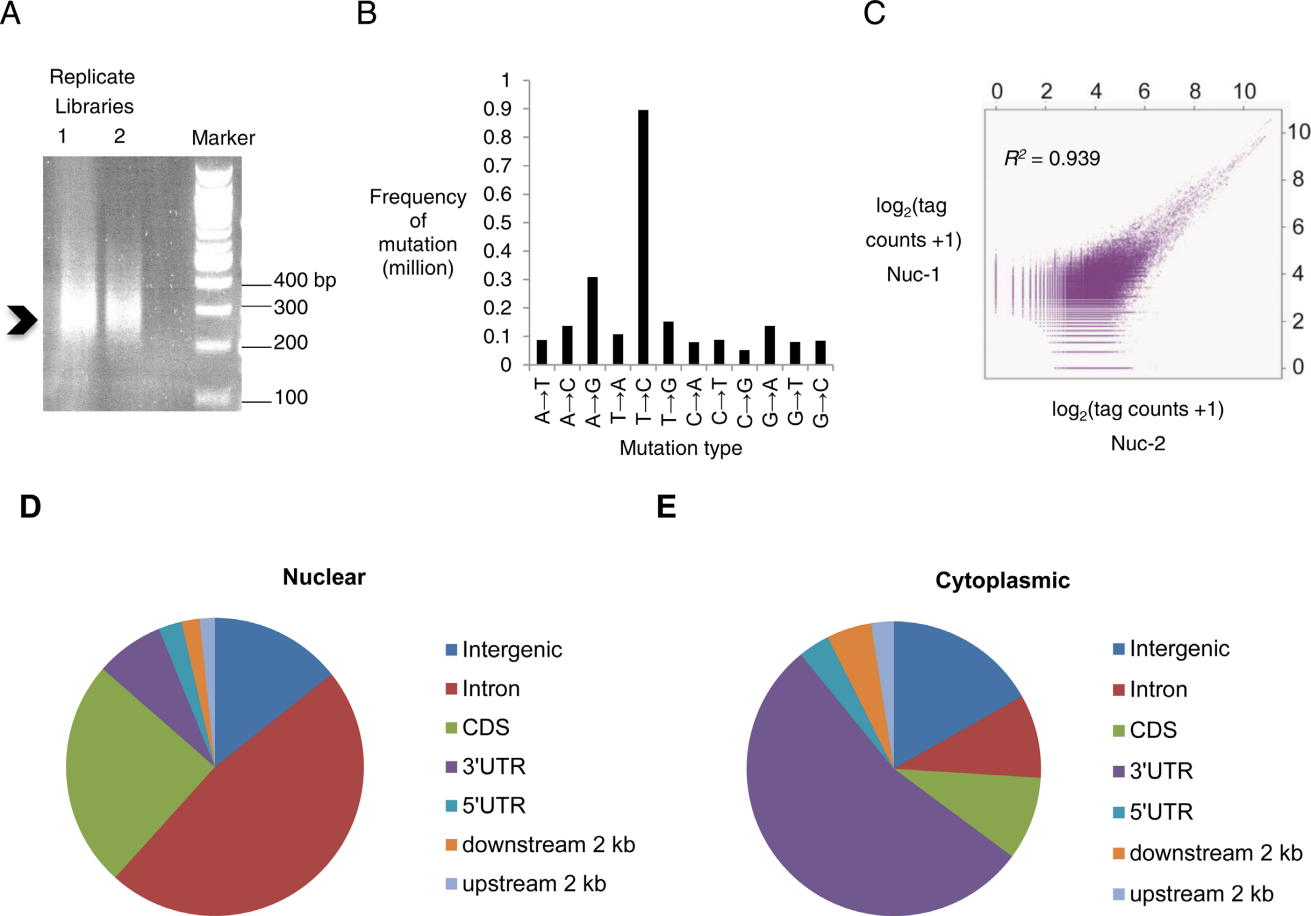
Preparation of RC-Seq libraries and analysis of PAR-CLIP derived binding sites. (**A**) RC-Seq libraries derived from Ago2 PAR-CLIP revealed on an agarose gel (1.5%), indicating a size to be 200–450 bp in length. (**B**) Detection of mutations in RC-Seq library derived from Ago2 PAR-CLIP, showing the expected dominance of T to C transition mutation from the incorporation of 4-SU. (**C**) Scatter plot showing the reproducibility of RC-Seq data from two biological nuclear replicates (Nuc-1 and Nuc-2). Coefficient of determination, *R*^2^ = 0.939. The unique tags from each replicate were clustered and the clusters were binned by a length of 10 bp. The natural logarithm transformed count data (plus 1) in each bin of a cluster are plotted to show the correlation between the replicates. (**D**) Genomic annotation of Ago2 significant binding clusters identified in two nuclear samples (Nuc-1 and Nuc-2). (**E**) Genomic annotation of Ago2 significant binding clusters identified in three cytoplasmic samples. MiClip program was used in searching the significant clusters in aligned datasets.

After observing the poor outcome of library generation based on intermolecular ligation between RNA and adaptors, we applied our RC-Seq method to prepare libraries. After reverse transcription, 3′ end extension, and PCR amplification we obtained products ranging in size from 200 to 450 bp for replicate libraries (Figure 8A). The size was 200–450 bp rather than the 20–50 nucleotides in the pool of captured RNA because multiple copies of the RNA sequences are incorporated into each amplified cDNA molecule (Figure 1).

The 200–450 bp range is typical for long RNA-Seq library and allowed the use of paired-end sequencing. Another advantage of using 200–450 bp fragments is that the crude RC-Seq library of CLIP-Seq RNA can be purified with AMPure XP magnetic beads. Use of these beads avoids the laborious and low efficiency PAGE purification step required for standard small RNA-Seq methods and further improves the efficiency of the protocol.

We sequenced the nuclear Ago2 PAR-CLIP library derived from our RC-Seq protocols and analyzed the results. The aligned sequencing data showed a dominant T to C mutation, consistent with incorporation of 4-SU and successful adaptation of the PAR-CLIP protocol to RC-Seq (Figure 8B) (14). We found that 10–12% of uniquely aligned reads had T to C mutations, a typical rate observed during PAR-CLIP. We obtained an average of 40 million raw reads and 9 million uniquely aligned reads from duplicate experiments. Using MiClip (23), a program weighing the T to C mutations and optimized to search for significant RNA clusters from CLIP-Seq datasets, we identified 7839 clusters from two biological replicates.

The sequencing data were reproducible between duplicate determinations with a strong concordance between grouped aligned reads (Figure 8C). Of 7839 total clusters, 7187 appeared in both replicates and the coefficient of determination was *R*^2^ = 0.94. Subsequent genomic annotation shows that >50% of clusters are localized within intronic regions (Figure 8D). This data is in a sharp contrast to data from three cytoplasmic samples that showed most clusters within the 3′-untranslated region of mRNAs (Figure 8E), likely reflecting differing roles for cytoplasmic and nuclear RNAi (13,14). The data variance in three cytoplasmic samples was also determined, with 0.82 < *R*^2^ < 0.94 between any two replicates (Supplementary Figure S5).

It has become clear that background in PAR-CLIP experiments needs to be taken into account for proper interpretation of data (31). To filter out the background clusters in nuclear Ago2 samples, we cut out a gel slice corresponding to ∼110 kDa band from the polyacrylamide gel from the IgG control sample. We subsequently sequenced the associated RNAs using the same methods we had applied to the analysis of nuclear Ago2 samples. By running the MiClip computational package, we obtained 29 significant binding sites. Genomic annotation analysis (Supplementary Figure S6) indicated that most of these 29 binding sites map to intergenic regions. These results are unlike the significant clusters from nuclear or cytoplasmic samples that mainly map to protein-encoding regions, promoters, or introns. 26 out of the 29 clusters from the IgG sample overlap with binding sites from Ago2 nuclear samples, but these numbers are small compared to the total number of significant binding sites identified for Ago2 nuclear samples. These background clusters can be filtered out to remove false candidates and facilitate subsequent experimental validation.

To further evaluate the quality of our data, we examined the overlap between the nuclear Ago2 binding sites detected by RC-Seq and those determined by other methods using Ago2 PAR-CLIP. There are no publically available data specifically on nuclear Ago2 PAR-CLIP for comparison, but we found six publically accessible Ago2 PAR-CLIP datasets generated from using whole cell lysate (14,32–36). The binding sites identified in these studies vary, with the number of significant binding sites ranging from 2000 to 44 000. Because all six datasets were generated using whole cell lysate for immunoprecipitation, it is difficult to make a direct comparison between these datasets and ours. In addition, these studies used different anti-Ago2 antibodies and cell lines.

Notwithstanding these differences, we carried out a comparison to determine overlap in potential binding sites between these previous studies and our own (Supplementary Figure S7A), and the previous studies with one another (Supplementary Figure S7B). We found that ≤10% of the clusters overlapped between the individual whole cell datasets and our nuclear data (Supplementary Figure S7A). The overlapping percentage among any two of the six datasets ranged from 15 to 45% (Supplementary Figure S7B). When comparing our cytoplasmic Ago2 datasets to those six published datasets from whole cell lysate, similar overlapping cluster percentage (15–50%) was obtained (data not shown). These comparisons suggest that our RC-Seq method identifies many of the same cytoplasmic signifcant clusters as had been observed previously, but that the identities of cytoplasmic and nuclear clusters are substantially different.

**Figure 9.**
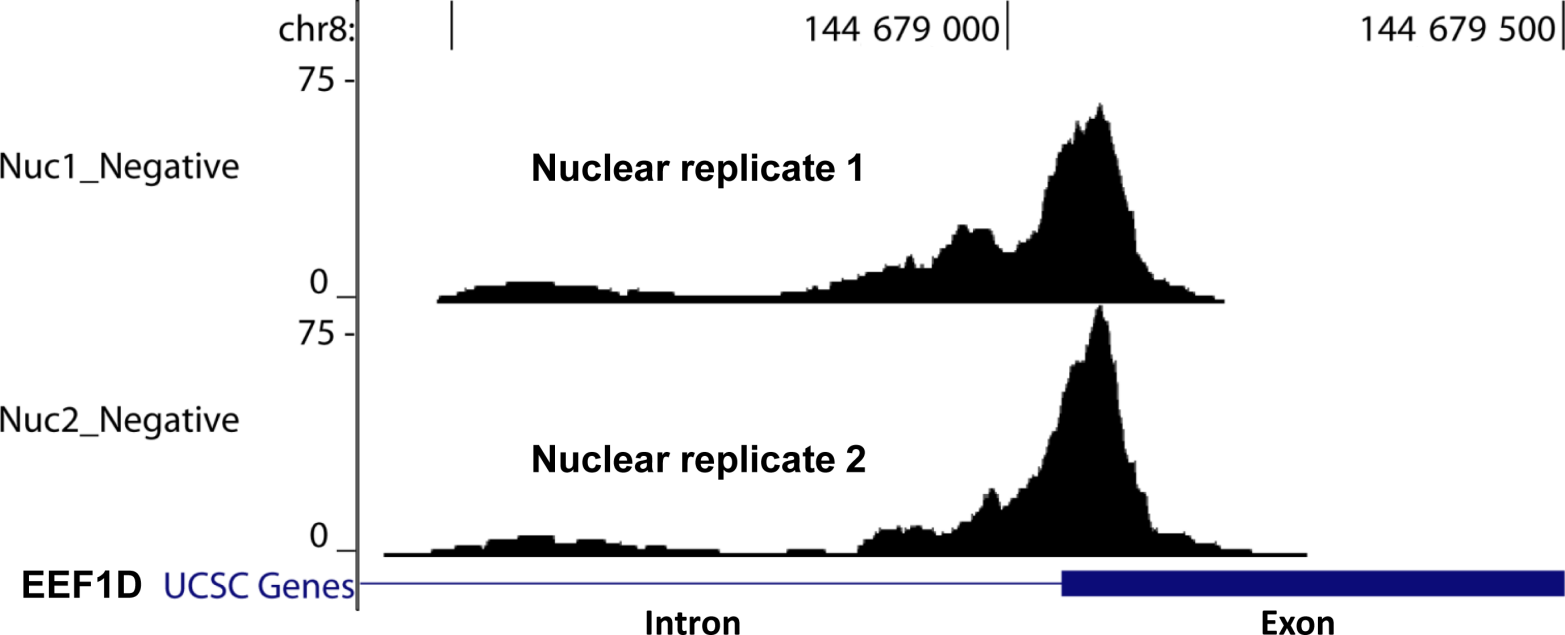
A significant Ago2 binding cluster lying at a EEF1D splicing junction identified from two nuclear PAR-CLIP sequencing datasets.

Ongoing studies have analyzed data from ten additional RC-Seq experiments and have shown similar sensitivity and reproducibility from nuclear, chromatin, and cytoplasmic fractions (Chu, unpublished). Analysis of duplicate read clusters from independent experiments has shown high reproducibility both in the breadth and shape of the clustered reads relative to the transcript targets. For example, at the intron-exon junction for the gene EEF1D we observed almost perfect overlap in both read location and distribution between two independent replicate experiments (Figure 9).

## DISCUSSION

RNA-Seq is a valuable technology and is becoming increasingly important as costs decrease and computational analysis becomes more accessible. Challenges to even more widespread use include the need to analyze small sample quantities with greater sensitivity. Meeting these challenges will require innovative approaches to library preparation.

Standard methods for sequencing small RNAs (<200 bases) are limited by the need to employ sequential intermolecular RNA and adaptor ligations (9) and sequencing less than 1 μg of total RNA input is often not recommended (http://support.illumina.com/sequencing). Insufficient amounts of available RNA may be a significant obstacle for some investigations of small RNAs including miRNAs, piRNAs, snoRNAs, extracellular small RNAs, and RNAs fragmented generated by approaches like CLIP.

### RC-Seq

We have shown that, by combining intramolecular RNA circularization and RNA-based rolling circle amplification (RCA), small RNA sequencing sensitivity can be greatly increased. In addition, using RCA (the cDNA molecules are significantly larger than the original RNA templates) makes it feasible to exploit a library purification approach that uses magnetic beads—an approach commonly used to purify long mRNA-Seq libraries. Finally, the first step is the RNA self-circularization carried out at 60°C by a thermostable Circligase II. The higher temperature for circularization reaction reduces interactions among RNA template molecules, making more sequences accessible to analysis.

RNA circularization followed by RCA has been used to detect specific miRNAs with quantitative PCR (25). This method, however, is not applicable to small RNA sequencing. During our preparation of this manuscript, Acevedo et al. reported a new long RNA-seq method, CirSeq, which is based on RNA circularization and RCA using random hexamers (37). In CirSeq, long RNA transcripts are first partially fragmented and only 80–90 nt RNA fragments are kept for library preparation. CirSeq, however, needs at least 1 μg of long RNA for library preparation. In contrast, RC-Seq is developed for small RNA sequencing with RNA size ranging from 20 nt to 100 nt. RC-Seq requires only subnanogram amounts of small RNA as input. To directly compare the sensitivity between CirSeq and RC-Seq would require more sequencing data, however, we do note that different types of enzymes are used at some of the key steps between these two methods. For example, RC-Seq uses Superscript II as reverse transcriptase for RCA, rather than Superscript III used by CirSeq. We have found that with small circular RNA (such as 20–60 nt RNA circles) as template, Superscript II is a more efficient enzyme than Superscript III (data not shown).

To achieve strand-specific sequencing, RC-Seq first uses tagged random primers to generate the first strand cDNA during RCA, rather than random hexamers used in CirSeq or other traditional long RNA-Seq methods. This first strand cDNA is further extended with a DNA polymerase and 3′-end blocked tagged random primers so that a PCR can be applied directly on the first strand of cDNA and original RNA strand information may be inferred from the sequencing reads. Tagged random priming has been used in efficient long RNA sequencing (7) and genomic DNA-Seq (38).

One recent report described an ultrasensitive ligation free RNA-Seq method called CATS (Capture and Amplification by Tailing and Switching) which can sequence as low as 100 pg of small (or long) RNA or DNA (39). In CATS the RNA undergoes polyadenylation first, followed by cDNA synthesis with tagged poly-dT primer. At the end of RT step, a template-switching strategy is used to incorporate a binding site for PCR primers. The strength of the method is its high sensitivity. Our approach complements CATS by avoiding a polyadenylation step and using DNA polymerase to extend the cDNA and permitting purification by magnetic beads.

When using less than 1ng of small RNA input for RC-Seq, extensive PCR amplification (more than 20 cycles) is often needed to generate sufficient DNA for sequencing. Thus, non-uniform amplification of cDNA molecules could become an issue. A few publications have explored the use of random sequences to correct for PCR-generated artifacts (40–43). In the case of RC-Seq, a 4-base random sequence can be incorporated into the primer used in reverse transcription step, such as 5′-GACGTGTGCTCTTCCGATCT**NNNN**GNNNNNN, to enable collapsing the observed read count to the number of observed random sequences. Thus we may be able to distinguish between reads that are PCR duplicates (from the same cDNA) and those that represent distinct cDNAs.

### Using RC-Seq

Our laboratory has a longstanding interest in the nuclear interactions between RNA and RNAi factors like argonaute and GW182. Relative to RNA from whole cells, nuclear RNA is scarce and difficult to obtain in quantities typically used for RNA-Seq. For CLIP-Seq, the RNA being analyzed is also limited by the efficiency of the crosslinking and immunoprecipitation steps. When attempting CLIP-Seq using isolated nuclear RNA and standard ligation-based methods, we failed to prepare a library that could serve as a basis for sequencing. We concluded that improving library preparation methods would be necessary to accomplish the investigation. Using RC-Seq, we were able to identify more than 7000 significant Ago2 binding clusters in cell nucleus, further highlighting the high sensitivity of RC-Seq.

For a sequencing method to be used widely, it must show good reproducibility in generating sequencing libraries. We obtained a coefficient of determination *R*^2^ = 0.94 for two nuclear replicates. For three cytoplasmic sequencing datasets a 0.82 < *R*^2^ < 0.94 was determined between any two replicates. By carrying out an Argonaute HITS-CLIP using whole-cell lysate and a ligation based library preparation approach, Chi *et al*. reported a 0.80 < *R*^2^ < 0.83 between datasets (13). In another related publication, Haecker *et al*. reported a *R*^2^ = 0.88 (44). Our results indicate that the RC-Seq method is highly reproducible and generates library with variance within the acceptable range.

It is not surprising to see limited overlap between our nuclear Ago2 binding sites with those published datasets. The published Ago2 PAR-CLIP were carried out using whole-cell lysate for the immunoprecipitation, likely with an emphasis on cell soluble fraction. When preparing nuclear samples, we purified nuclei first, followed by sonication and DNase I treatment to remove chromosome DNA, so that maximum amount of Ago2-associated RNA could be recovered. These steps were not applied by prior publications when harvesting the whole cell lysate. In addition, the difference in cell line being studied and different types of anti-Ago2 antibodies being used may also contribute to the observed difference on cluster locations among these samples.

In summary, our RC-Seq data on nuclear Ago2 associations suggest previously unknown interactions between RNA and RNAi factors in cell nuclei and will guide detailed investigations into the natural function of nuclear RNAi and its potential impact on splicing and transcription. More generally, successful application of RC-Seq to PAR-CLIP indicates that intramolecular circularization during library preparation can facilitate access to sequencing data from samples where amounts of RNA are limiting and structures of RNA hinder intermolecular ligation. Rare RNA sequences can be analyzed with better coverage, allowing more accurate characterization of transcripts that may be present in only a few copies per cell.

## SUPPLEMENTARY DATA

Supplementary Data are available at NAR Online.

SUPPLEMENTARY DATA
