# Supplementary material for: Intramolecular circularization increases efficiency of RNA sequencing and enables CLIP-Seq of nuclear RNA from human cells
Source: Nucleic Acids Res. 2015 Mar 26;43(11):e75. doi: 10.1093/nar/gkv213 (PMC4477644; doi:10.1093/nar/gkv213)
Supplement: SUPPLEMENTARY DATA [file supp_43_11_e75__index.html]

Intramolecular circularization increases efficiency of RNA sequencing and enables CLIP-Seq of nuclear RNA from human cells — Intramolecular circularization increases efficiency of RNA sequencing and enables CLIP-Seq of nuclear RNA from human cells — SUPPLEMENTARY DATA 

# Intramolecular circularization increases efficiency of RNA sequencing and enables CLIP-Seq of nuclear RNA from human cells

## SUPPLEMENTARY DATA

**Files in this Data Supplement:**

- SUPPLEMENTARY DATA
