# Supplementary material for: Intramolecular circularization increases efficiency of RNA sequencing and enables CLIP-Seq of nuclear RNA from human cells
Source: Nucleic Acids Res. 2015 Mar 26;43(11):e75. doi: 10.1093/nar/gkv213 (PMC4477644; doi:10.1093/nar/gkv213)
Supplement: SUPPLEMENTARY DATA [file supp_gkv213_nar-00016-met-g-2015-File003.pptx]

## Slide 1
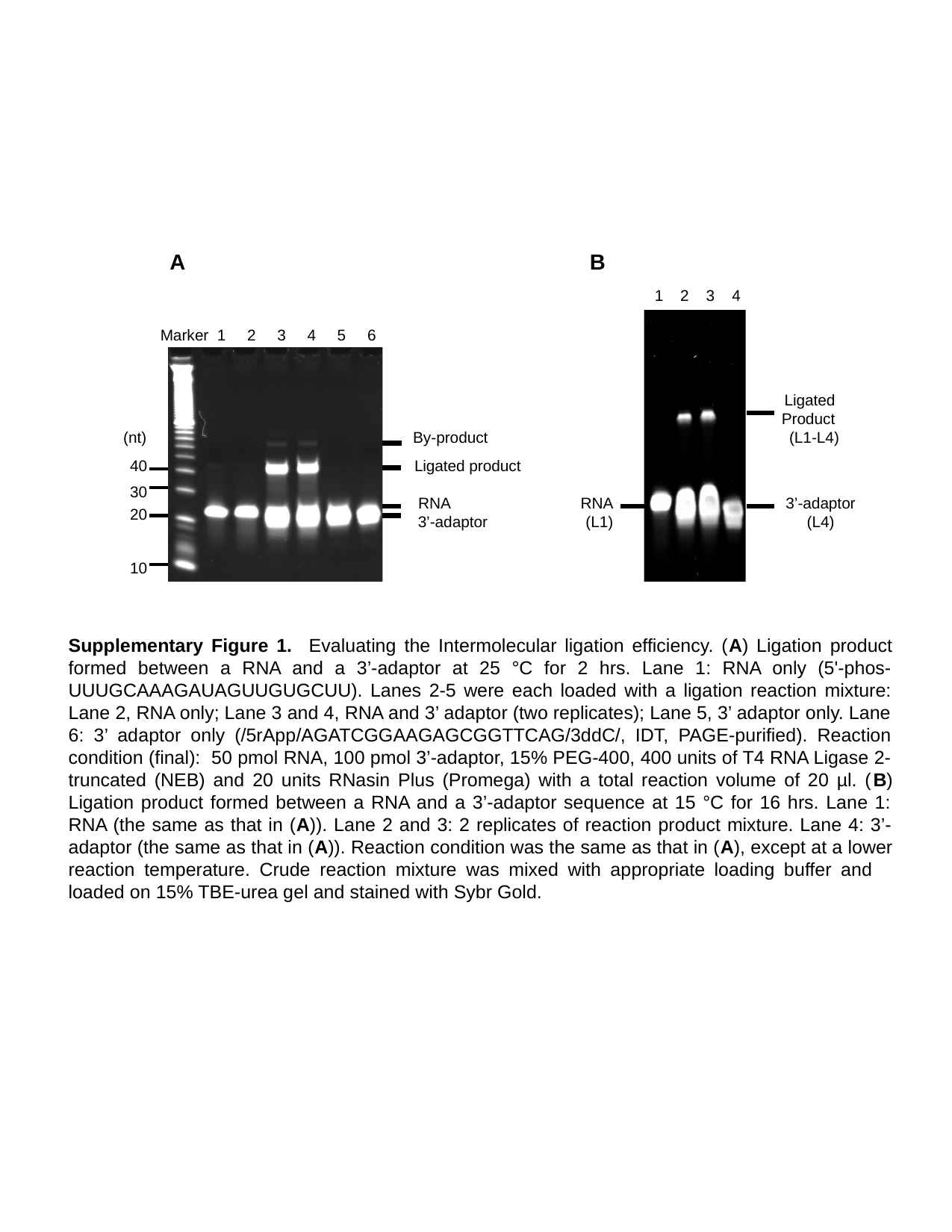

A
B
 1 2 3 4
Marker 1 2 3 4 5 6
Ligated
Product
(L1-L4)
(nt)
By-product
40
Ligated product
30
RNA
RNA
(L1)
3’-adaptor
(L4)
20
3’-adaptor
10
Supplementary Figure 1. Evaluating the Intermolecular ligation efficiency. (A) Ligation product formed between a RNA and a 3’-adaptor at 25 °C for 2 hrs. Lane 1: RNA only (5'-phos-UUUGCAAAGAUAGUUGUGCUU). Lanes 2-5 were each loaded with a ligation reaction mixture: Lane 2, RNA only; Lane 3 and 4, RNA and 3’ adaptor (two replicates); Lane 5, 3’ adaptor only. Lane 6: 3’ adaptor only (/5rApp/AGATCGGAAGAGCGGTTCAG/3ddC/, IDT, PAGE-purified). Reaction condition (final): 50 pmol RNA, 100 pmol 3’-adaptor, 15% PEG-400, 400 units of T4 RNA Ligase 2-truncated (NEB) and 20 units RNasin Plus (Promega) with a total reaction volume of 20 µl. (B) Ligation product formed between a RNA and a 3’-adaptor sequence at 15 °C for 16 hrs. Lane 1: RNA (the same as that in (A)). Lane 2 and 3: 2 replicates of reaction product mixture. Lane 4: 3’-adaptor (the same as that in (A)). Reaction condition was the same as that in (A), except at a lower reaction temperature. Crude reaction mixture was mixed with appropriate loading buffer and loaded on 15% TBE-urea gel and stained with Sybr Gold.

## Slide 2
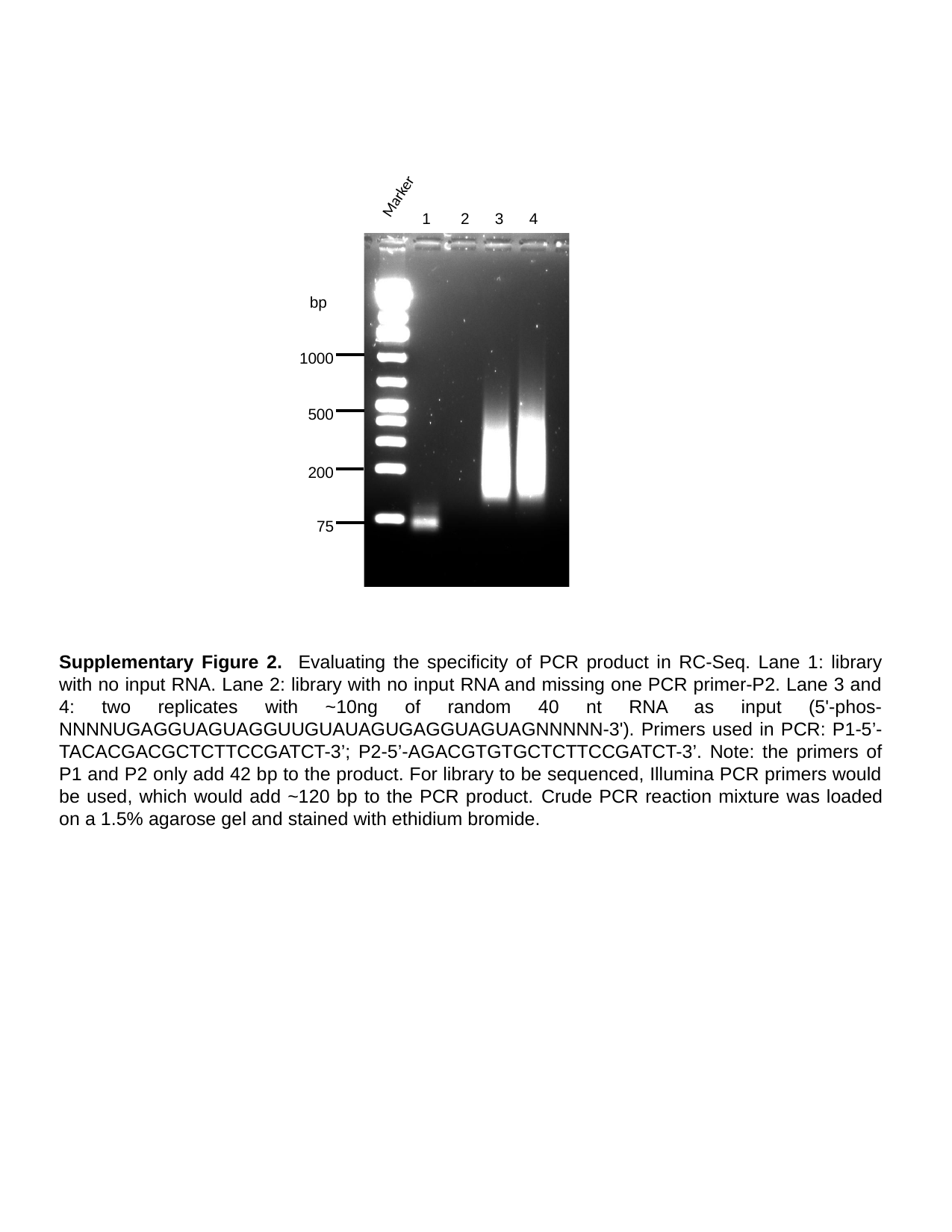

Marker
1 2 3 4
bp
1000
500
200
75
Supplementary Figure 2. Evaluating the specificity of PCR product in RC-Seq. Lane 1: library with no input RNA. Lane 2: library with no input RNA and missing one PCR primer-P2. Lane 3 and 4: two replicates with ~10ng of random 40 nt RNA as input (5'-phos-NNNNUGAGGUAGUAGGUUGUAUAGUGAGGUAGUAGNNNNN-3'). Primers used in PCR: P1-5’-TACACGACGCTCTTCCGATCT-3’; P2-5’-AGACGTGTGCTCTTCCGATCT-3’. Note: the primers of P1 and P2 only add 42 bp to the product. For library to be sequenced, Illumina PCR primers would be used, which would add ~120 bp to the PCR product. Crude PCR reaction mixture was loaded on a 1.5% agarose gel and stained with ethidium bromide.

## Slide 3
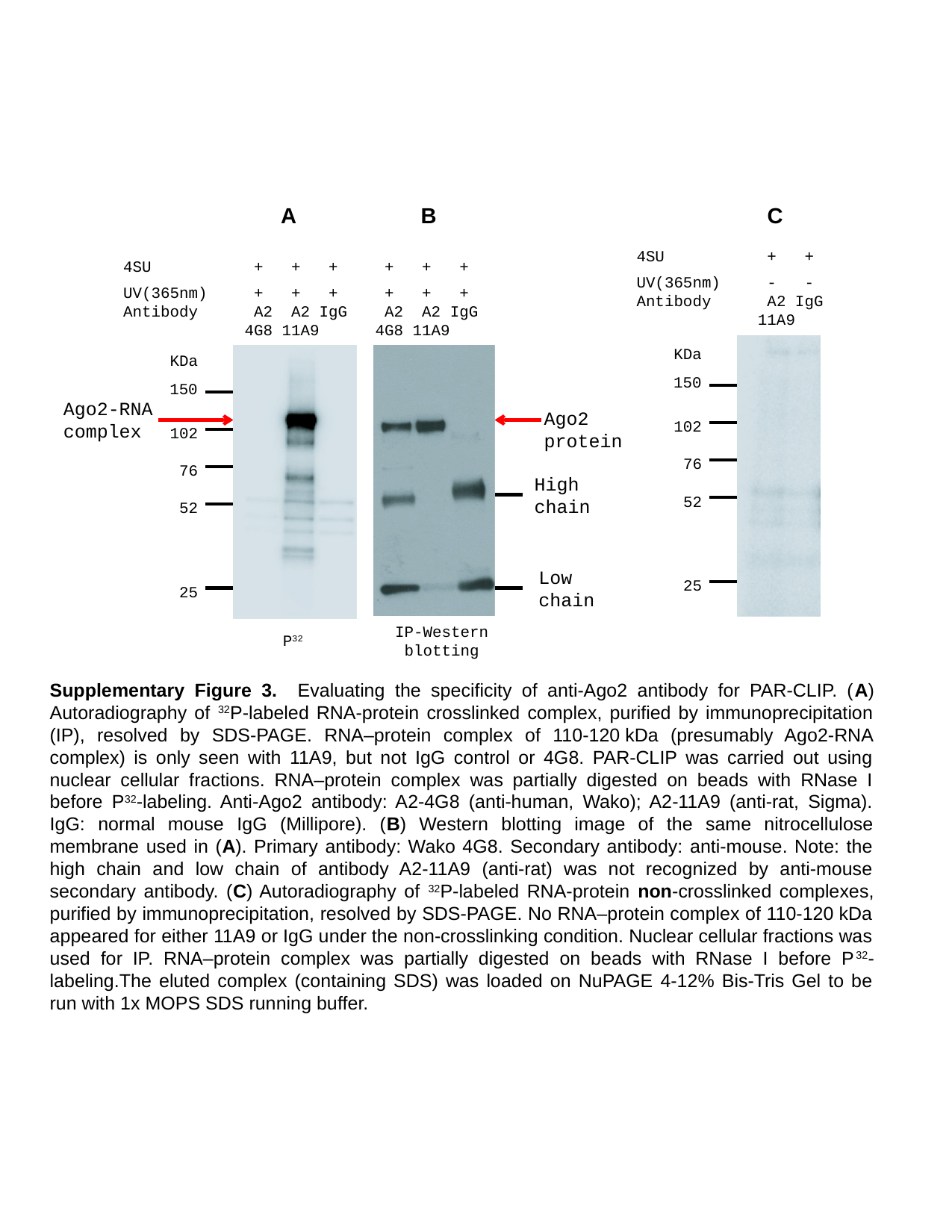

A
B
C
4SU + +
UV(365nm) - -
Antibody A2 IgG
 11A9
4SU + + + + + +
UV(365nm) + + + + + +
Antibody A2 A2 IgG A2 A2 IgG
 4G8 11A9 4G8 11A9
KDa
KDa
150
150
Ago2-RNA
complex
Ago2
protein
102
102
76
76
High
chain
52
52
Low
chain
25
25
IP-Western
blotting
P32
Supplementary Figure 3. Evaluating the specificity of anti-Ago2 antibody for PAR-CLIP. (A) Autoradiography of 32P-labeled RNA-protein crosslinked complex, purified by immunoprecipitation (IP), resolved by SDS-PAGE. RNA–protein complex of 110-120 kDa (presumably Ago2-RNA complex) is only seen with 11A9, but not IgG control or 4G8. PAR-CLIP was carried out using nuclear cellular fractions. RNA–protein complex was partially digested on beads with RNase I before P32-labeling. Anti-Ago2 antibody: A2-4G8 (anti-human, Wako); A2-11A9 (anti-rat, Sigma). IgG: normal mouse IgG (Millipore). (B) Western blotting image of the same nitrocellulose membrane used in (A). Primary antibody: Wako 4G8. Secondary antibody: anti-mouse. Note: the high chain and low chain of antibody A2-11A9 (anti-rat) was not recognized by anti-mouse secondary antibody. (C) Autoradiography of 32P-labeled RNA-protein non-crosslinked complexes, purified by immunoprecipitation, resolved by SDS-PAGE. No RNA–protein complex of 110-120 kDa appeared for either 11A9 or IgG under the non-crosslinking condition. Nuclear cellular fractions was used for IP. RNA–protein complex was partially digested on beads with RNase I before P32-labeling.The eluted complex (containing SDS) was loaded on NuPAGE 4-12% Bis-Tris Gel to be run with 1x MOPS SDS running buffer.

## Slide 4
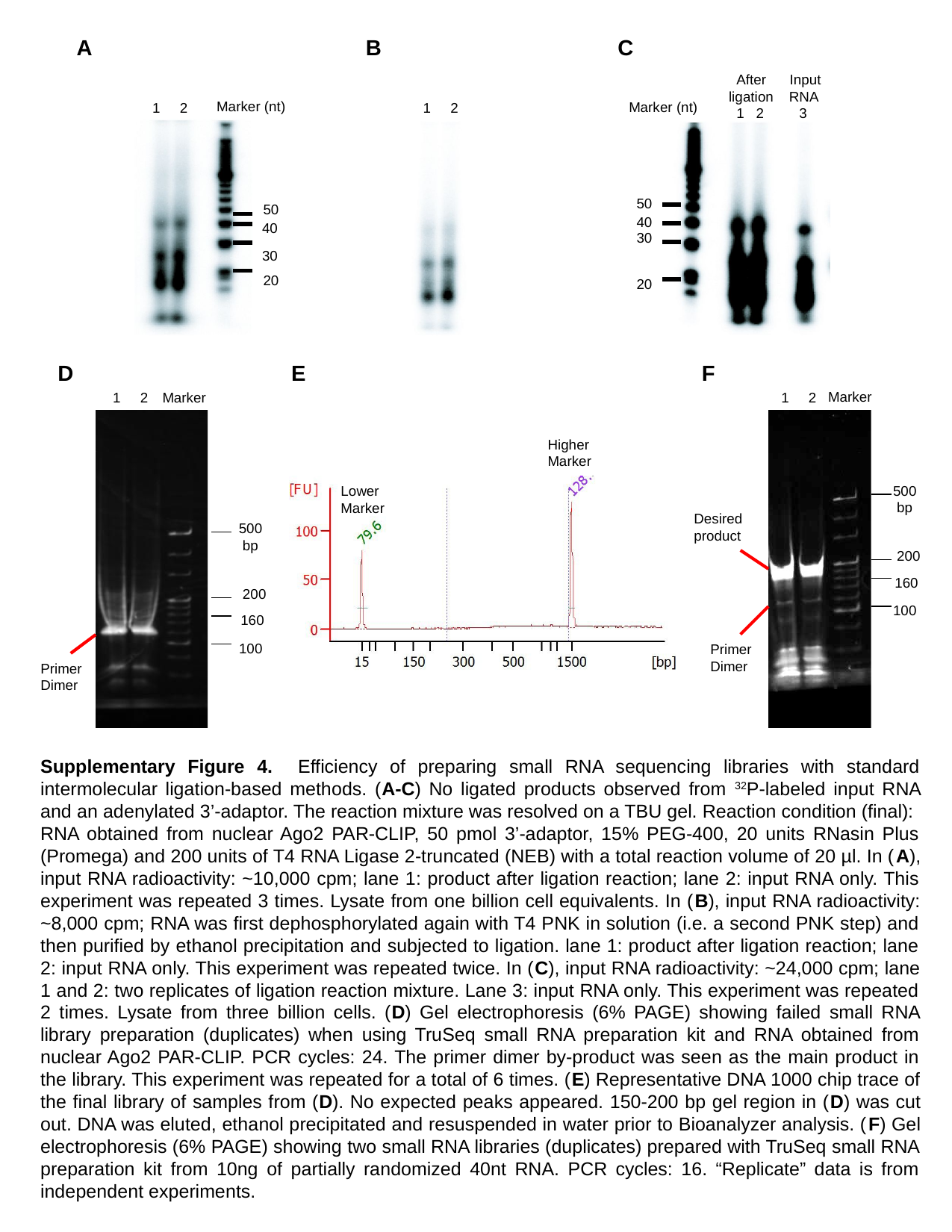

A
B
C
 After Input
ligation RNA
 1 2 3
Marker (nt)
Marker (nt)
1 2
1 2
50
50
40
40
30
30
20
20
D
E
F
Marker
1 2
1 2
Marker
Higher
Marker
500
 bp
Lower
Marker
Desired
product
500
 bp
200
160
200
100
160
100
Primer
Dimer
Primer
Dimer
Supplementary Figure 4. Efficiency of preparing small RNA sequencing libraries with standard intermolecular ligation-based methods. (A-C) No ligated products observed from 32P-labeled input RNA and an adenylated 3’-adaptor. The reaction mixture was resolved on a TBU gel. Reaction condition (final): RNA obtained from nuclear Ago2 PAR-CLIP, 50 pmol 3’-adaptor, 15% PEG-400, 20 units RNasin Plus (Promega) and 200 units of T4 RNA Ligase 2-truncated (NEB) with a total reaction volume of 20 µl. In (A), input RNA radioactivity: ~10,000 cpm; lane 1: product after ligation reaction; lane 2: input RNA only. This experiment was repeated 3 times. Lysate from one billion cell equivalents. In (B), input RNA radioactivity: ~8,000 cpm; RNA was first dephosphorylated again with T4 PNK in solution (i.e. a second PNK step) and then purified by ethanol precipitation and subjected to ligation. lane 1: product after ligation reaction; lane 2: input RNA only. This experiment was repeated twice. In (C), input RNA radioactivity: ~24,000 cpm; lane 1 and 2: two replicates of ligation reaction mixture. Lane 3: input RNA only. This experiment was repeated 2 times. Lysate from three billion cells. (D) Gel electrophoresis (6% PAGE) showing failed small RNA library preparation (duplicates) when using TruSeq small RNA preparation kit and RNA obtained from nuclear Ago2 PAR-CLIP. PCR cycles: 24. The primer dimer by-product was seen as the main product in the library. This experiment was repeated for a total of 6 times. (E) Representative DNA 1000 chip trace of the final library of samples from (D). No expected peaks appeared. 150-200 bp gel region in (D) was cut out. DNA was eluted, ethanol precipitated and resuspended in water prior to Bioanalyzer analysis. (F) Gel electrophoresis (6% PAGE) showing two small RNA libraries (duplicates) prepared with TruSeq small RNA preparation kit from 10ng of partially randomized 40nt RNA. PCR cycles: 16. “Replicate” data is from independent experiments.

## Slide 5
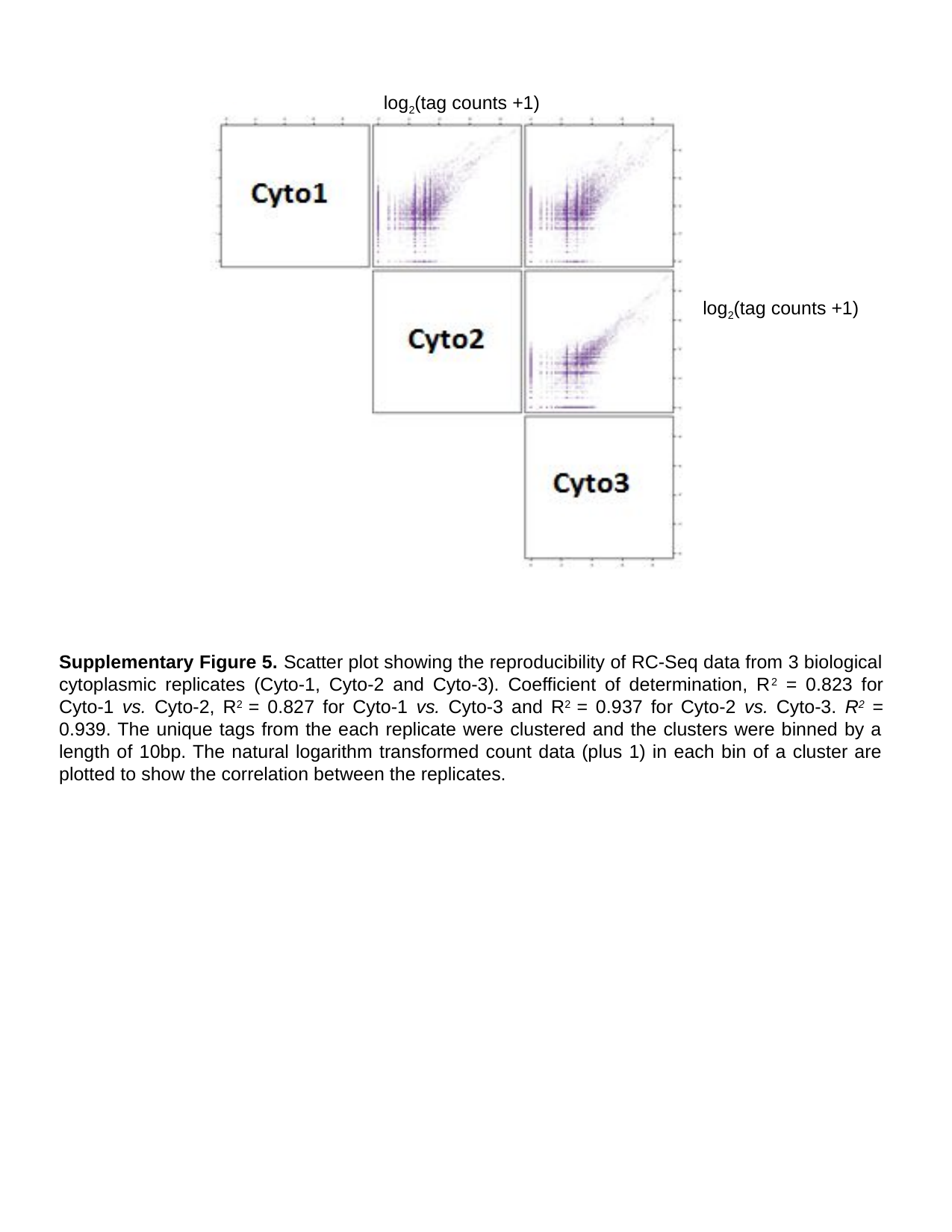

log2(tag counts +1)
log2(tag counts +1)
Supplementary Figure 5. Scatter plot showing the reproducibility of RC-Seq data from 3 biological cytoplasmic replicates (Cyto-1, Cyto-2 and Cyto-3). Coefficient of determination, R2 = 0.823 for Cyto-1 vs. Cyto-2, R2 = 0.827 for Cyto-1 vs. Cyto-3 and R2 = 0.937 for Cyto-2 vs. Cyto-3. R2 = 0.939. The unique tags from the each replicate were clustered and the clusters were binned by a length of 10bp. The natural logarithm transformed count data (plus 1) in each bin of a cluster are plotted to show the correlation between the replicates.

## Slide 6
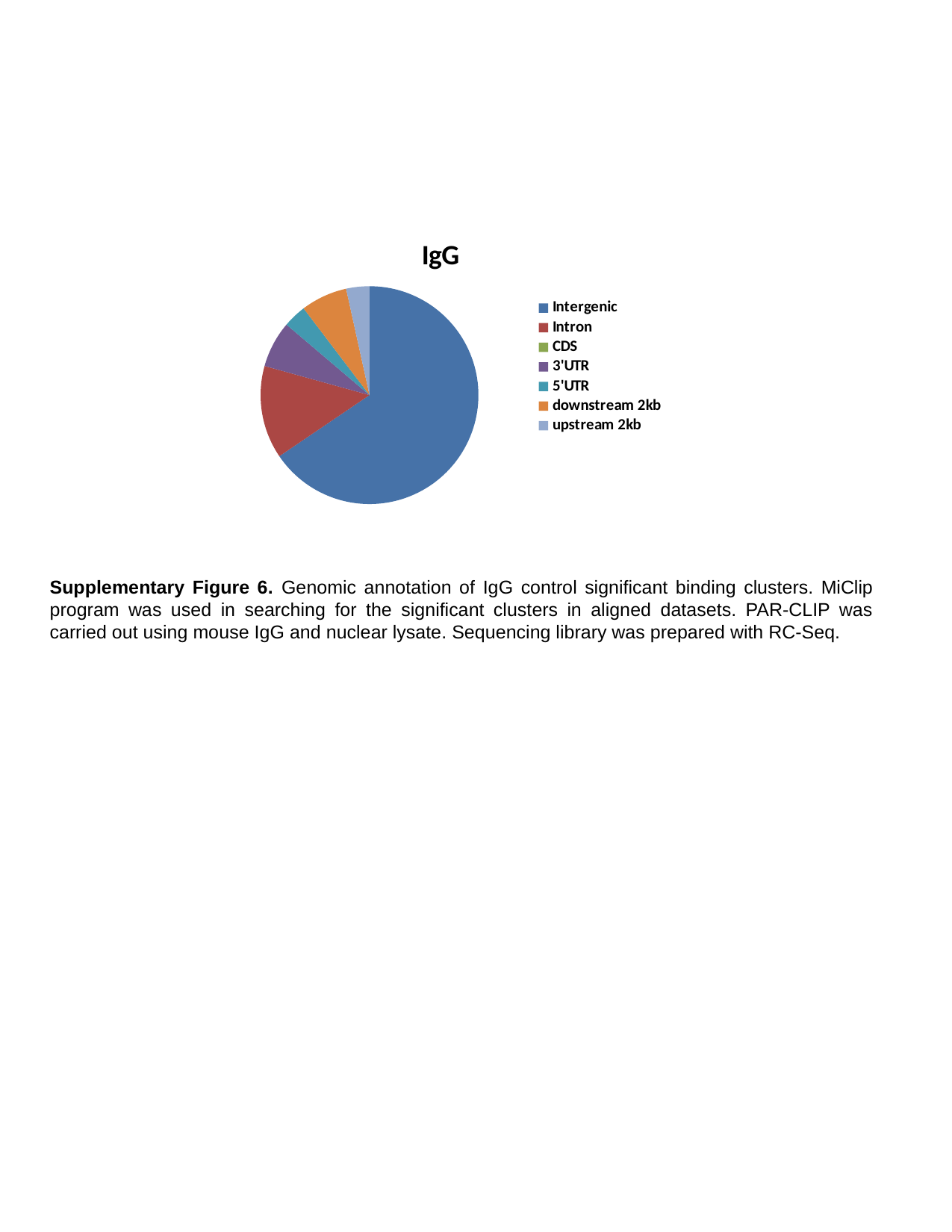

### Chart: IgG
| Category | |
|---|---|
| Intergenic | 19.0 |
| Intron | 4.0 |
| CDS | 0.0 |
| 3'UTR | 2.0 |
| 5'UTR | 1.0 |
| downstream 2kb | 2.0 |
| upstream 2kb | 1.0 |Supplementary Figure 6. Genomic annotation of IgG control significant binding clusters. MiClip program was used in searching for the significant clusters in aligned datasets. PAR-CLIP was carried out using mouse IgG and nuclear lysate. Sequencing library was prepared with RC-Seq.

## Slide 7
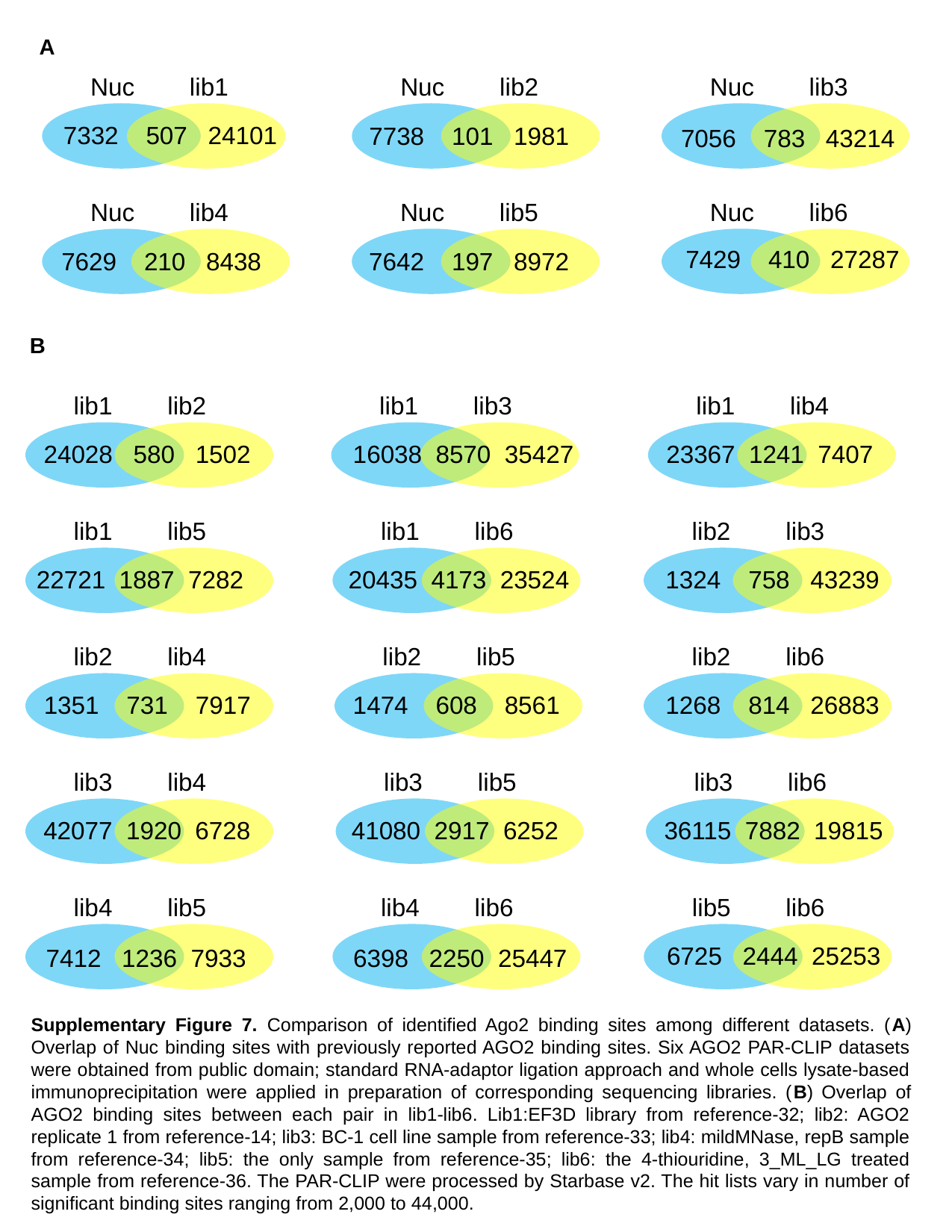

A
Nuc lib1
7332 507 24101
Nuc lib2
7738 101 1981
Nuc lib3
7056 783 43214
Nuc lib4
7629 210 8438
Nuc lib5
7642 197 8972
Nuc lib6
7429 410 27287
B
lib1 lib2
24028 580 1502
lib1 lib3
16038 8570 35427
lib1 lib4
23367 1241 7407
lib1 lib5
22721 1887 7282
lib1 lib6
20435 4173 23524
lib2 lib3
1324 758 43239
lib2 lib4
1351 731 7917
lib2 lib5
1474 608 8561
lib2 lib6
1268 814 26883
lib3 lib4
42077 1920 6728
lib3 lib5
41080 2917 6252
lib3 lib6
36115 7882 19815
lib4 lib5
 7412 1236 7933
lib4 lib6
 6398 2250 25447
lib5 lib6
 6725 2444 25253
Supplementary Figure 7. Comparison of identified Ago2 binding sites among different datasets. (A) Overlap of Nuc binding sites with previously reported AGO2 binding sites. Six AGO2 PAR-CLIP datasets were obtained from public domain; standard RNA-adaptor ligation approach and whole cells lysate-based immunoprecipitation were applied in preparation of corresponding sequencing libraries. (B) Overlap of AGO2 binding sites between each pair in lib1-lib6. Lib1:EF3D library from reference-32; lib2: AGO2 replicate 1 from reference-14; lib3: BC-1 cell line sample from reference-33; lib4: mildMNase, repB sample from reference-34; lib5: the only sample from reference-35; lib6: the 4-thiouridine, 3_ML_LG treated sample from reference-36. The PAR-CLIP were processed by Starbase v2. The hit lists vary in number of significant binding sites ranging from 2,000 to 44,000.
